# Increased Abundance of Nuclear HDAC4 Impairs Neuronal Development and Long-Term Memory

**DOI:** 10.3389/fnmol.2021.616642

**Published:** 2021-03-30

**Authors:** Patrick Main, Wei Jun Tan, David Wheeler, Helen L. Fitzsimons

**Affiliations:** ^1^Biochemistry, Biotechnology and Biomedical Science Group, School of Fundamental Sciences, Massey University, Palmerston North, New Zealand; ^2^NSW Department of Primary Industries, Orange, NSW, Australia

**Keywords:** HDAC4, histone deacetylase, memory, neuron, Alzheimer, *Drosophila*, MEF2

## Abstract

Dysregulation of the histone deacetylase HDAC4 is associated with both neurodevelopmental and neurodegenerative disorders, and a feature common to many of these disorders is impaired cognitive function. HDAC4 shuttles between the nucleus and cytoplasm in both vertebrates and invertebrates and alterations in the amounts of nuclear and/or cytoplasmic HDAC4 have been implicated in these diseases. In *Drosophila*, HDAC4 also plays a critical role in the regulation of memory, however, the mechanisms through which it acts are unknown. Nuclear and cytoplasmically-restricted HDAC4 mutants were expressed in the *Drosophila* brain to investigate a mechanistic link between HDAC4 subcellular distribution, transcriptional changes and neuronal dysfunction. Deficits in mushroom body morphogenesis, eye development and long-term memory correlated with increased abundance of nuclear HDAC4 but were associated with minimal transcriptional changes. Although HDAC4 sequesters MEF2 into punctate foci within neuronal nuclei, no alteration in MEF2 activity was observed on overexpression of *HDAC4*, and knockdown of *MEF2* had no impact on long-term memory, indicating that HDAC4 is likely not acting through MEF2. In support of this, mutation of the MEF2 binding site within HDAC4 also had no impact on nuclear HDAC4-induced impairments in long-term memory or eye development. In contrast, the defects in mushroom body morphogenesis were ameliorated by mutation of the MEF2 binding site, as well as by co-expression of *MEF2* RNAi, thus nuclear HDAC4 acts through MEF2 to disrupt mushroom body development. These data provide insight into the mechanisms through which dysregulation of HDAC4 subcellular distribution impairs neurological function and provides new avenues for further investigation.

## Introduction

Dysregulation of the histone deacetylase HDAC4 results in impairments in neuronal development and formation of long-term memories in the *Drosophila* brain (Fitzsimons et al., 2013). Similarly in vertebrates, altered expression or subcellular distribution of HDAC4 is associated with both neurodevelopmental and neurodegenerative disorders, including 2q37 syndrome (previously called brachydactyly mental retardation syndrome) (Williams et al., 2010; Morris et al., 2012; Villavicencio-Lorini et al., 2013), CDKL5 disorder (Trazzi et al., 2016), Alzheimer’s disease (Cao et al., 2008; Herrup et al., 2013; Shen et al., 2016), autism (Williams et al., 2010; Pinto et al., 2014). Huntington’s disease (Mielcarek et al., 2013a) and ataxia telangiectasia (Li et al., 2012; Herrup et al., 2013; Shen et al., 2016). Impaired cognitive function is a feature common to all of these disorders. HDAC4 shuttles between the nucleus and cytoplasm in both vertebrates and invertebrates (Chawla et al., 2003; Fitzsimons et al., 2013) and alterations in the amounts of nuclear and/or cytoplasmic HDAC4 have been implicated in these diseases (Cao et al., 2008; Sando et al., 2012; Herrup et al., 2013; Mielcarek et al., 2013a, 2015; Shen et al., 2016). A frameshift mutation that results in nuclear accumulation of HDAC4 also results in features consistent with 2q37 deletion syndrome (Williams et al., 2010) and expression of the corresponding mouse variant of *HDAC4* in the mouse brain causes deficits in spatial learning and memory (Sando et al., 2012). This may be initially surprising given that this mutant lacks a deacetylase domain, however, HDAC4 is catalytically inactive in vertebrates and there are no global changes in histone acetylation resulting from knockout of *HDAC4* in mice (Mielcarek et al., 2013b). Nuclear accumulation of HDAC4 has been observed in human Alzheimer’s disease post-mortem brains as well as the brains of Alzheimer’s mice with the abundance of nuclear HDAC4 correlating with increased clinical dementia scores in humans (Herrup et al., 2013; Shen et al., 2016), indicating that excess abundance of nuclear HDAC4 impairs cognitive function.

Nuclear import of HDAC4 requires a nuclear localization signal as well as binding of the transcription factor MEF2. Mutation of leucine 175 to alanine (L175A) within the MEF2 binding region of HDAC4 prevents MEF2 binding and results in cytoplasmic accumulation of HDAC4 (Wang and Yang, 2001). Nuclear export is regulated *via* activity-dependent CaM kinase-mediated phosphorylation of conserved serine residues S246, S467, and S623, which allows binding of the chaperone 14-3-3ζ (Grozinger and Schreiber, 2000; McKinsey et al., 2000; Wang et al., 2000; Wang and Yang, 2001; Zhao et al., 2001), and export from the nucleus (McKinsey et al., 2001). Mutation of these residues to alanine (3SA) results in nuclear retention (Chawla et al., 2003; Sando et al., 2012; Schlumm et al., 2013). In the nucleus, HDAC4 has been identified as a transcriptional regulator that represses transcription factors such as MEF2 (Miska et al., 1999). MEF2 family members promote neuronal survival and regulate both memory formation and dendrite morphogenesis (Flavell et al., 2006; Shalizi et al., 2006; Cole et al., 2012). Furthermore, they regulate multiple synapse-associated genes, and promote the development of inhibitory synapses while repressing excitatory synapse development (Barbosa et al., 2008).

*Drosophila* HDAC4 (DmHDAC4) shares 57% amino acid identity and 84% similarity to human HDAC4 (hHDAC4) across the deacetylase domain-containing C terminus, and 35% identity and 59% similarity across the whole protein and it is the sole Class IIa HDAC in *Drosophila*. The N-terminal elements critical for function and regulation of subcellular localization are conserved in DmHDAC4, including the MEF2 binding motif, serine residues for 14-3-3 mediated nuclear export, and a putative nuclear import signal (Fitzsimons et al., 2013). We previously found that overexpression of wild-type *HDAC4* in the adult mushroom body, a critical structure for formation of associative memory in *Drosophila* (McBride et al., 1999) resulted in impaired long-term memory (LTM) in a *Drosophila* model of courtship memory. The LTM deficits that we observed were specific to LTM as short-term memory and courtship activity were unaffected (Fitzsimons et al., 2013). This suggested that HDAC4 facilitates altered expression of genes that are required for LTM, however, RNA-seq on *Drosophila* heads overexpressing *HDAC4* in the brain revealed very few changes (Schwartz et al., 2016). Moreover, it is likely that these effects are deacetylase independent (DmHDAC4 is catalytically active) as overexpression of an *HDAC4* variant with a H968A substitution that disrupts deacetylase activity (Miska et al., 1999) also caused severe impairment of LTM (Fitzsimons et al., 2013). In addition, knockdown of *HDAC4* in the mushroom body of adult flies caused a similar phenotype, with male flies unable to form long-term memories, supporting the hypothesis that HDAC4 is a repressor of memory when in excess but also plays an essential role in normal LTM. We also carried out a screen for genes that interact genetically with *HDAC4* in eye development, and identified genes encoding proteins both that interact with and/or regulate the actin cytoskeleton and influence neuronal growth, as well as transcriptional machinery and genes involved in SUMOylation (Schwartz et al., 2016). Despite these advances, the specific mechanisms through which HDAC4 acts are still unclear, and complicating our understanding is that nuclear accumulation of HDAC4 goes hand-in-hand with cytoplasmic depletion, making it unclear whether cytoplasmic HDAC4 is required for learning and memory or whether it is merely being sequestered outside of the nucleus.

Here we aimed to further investigate the role of HDAC4, specifically to determine a mechanistic link between HDAC4 subcellular distribution, transcriptional changes and neuronal dysfunction. Mutant variants of HDAC4 that were sequestered in the nucleus or cytoplasm were compared to wild-type *Drosophila* and human HDAC4 to untangle the separate roles of nuclear and cytoplasmic HDAC4 in development and memory, as well as directly comparing the activity of *Drosophila* and human HDAC4s. Both mammalian HDAC4 and DmHDAC4 have been demonstrated to repress memory in their respective models, however, until now their subcellular distribution and respective roles in neurons have not been directly compared.

## Results

### Characterization of Expression and Subcellular Distribution in the Mushroom Body

To characterize the contribution of subcellular pools of HDAC4 to the previously observed impairments in neuronal function, we employed transgenic flies expressing the hHDAC4 mutants that are sequestered in the nucleus or cytoplasm. Nuclear and cytoplasmically restricted hHDAC4 mutants have been previously characterized (Cohen et al., 2007; Chen and Cepko, 2009); *3SA* encodes a nucleus-sequestered variant of hHDAC4 with three serine to alanine mutations that prevent nuclear export, and *L175A* encodes a cytoplasm-sequestered variant that harbors a leucine to alanine mutation that prevents nuclear import.

**FIGURE 1:**
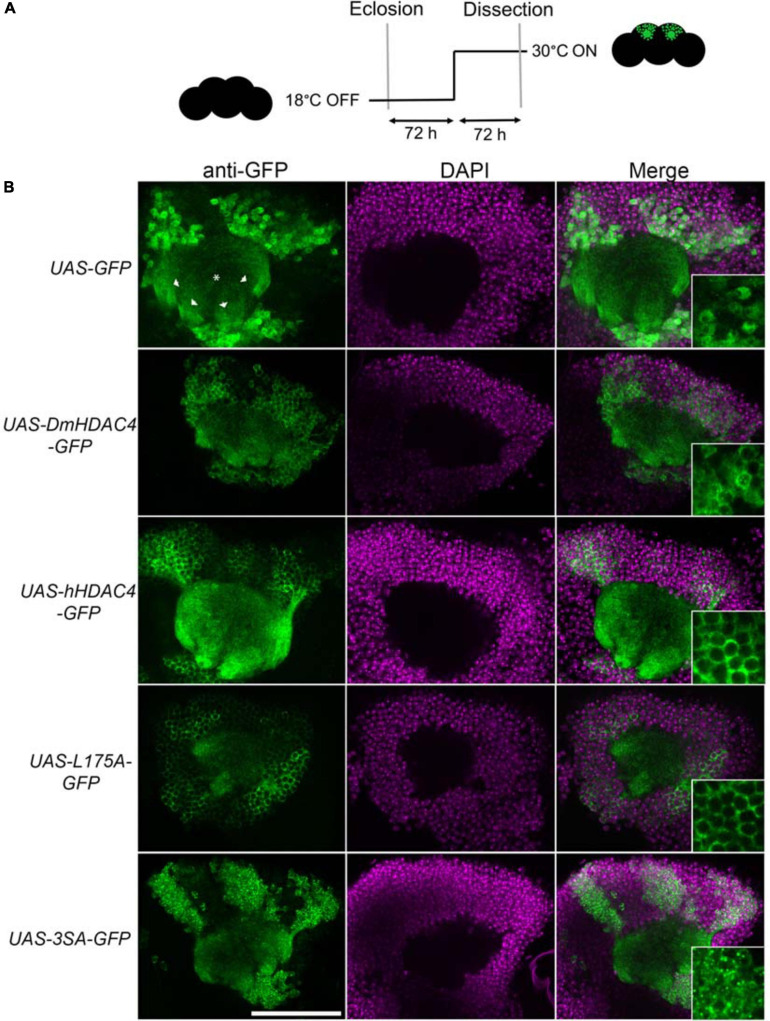
Expression and subcellular localization of GFP-tagged HDAC4 constructs in the mushroom body. **(A)** Schematic diagram describing the protocol for induction of expression in the adult mushroom body. All genotypes were generated by crossing *OK107-GAL4; tubP-GAL80_ts_* females to males carrying the indicated UAS-transgene. All *HDAC4* transgenes were fused in frame to GFP. Flies were raised at 18°C, at which temperature GAL80 represses GAL4, until after eclosion when the temperature was raised to 30°C. At this temperature GAL80 is inactivated, allowing GAL4 to induce transgene expression. Brains were dissected after 72 h at 30°C. **(B)** Whole-mount brains were subjected to immunohistochemistry with anti-GFP (green) and counterstained with DAPI (magenta). Optical sections (1 μm) through the calyx of the mushroom body are shown. Asterisks label the calyx and arrowheads indicate the four bundles of Kenyon cell axons that originate in the calyx and project anteriodorsally to form the lobes. Scale bar = 40 μm. Insets in the right panels are a 4× magnification of cell bodies.

Prior to functional analyses, the subcellular distribution of wild-type hHDAC4 and the mutants was compared to that of wild-type DmHDAC4. Expression was driven in the mushroom body with the *OK107-GAL4* driver (Connolly et al., 1996; Aso et al., 2009) and induced in adult flies *via* temperature-dependent manipulation of a temperature-sensitive mutant of GAL80 (GAL80ts). GAL4 is repressed by GAL80ts at the permissive temperature of 18°C, whereas when the temperature is raised to 30°C, GAL80ts is no longer able to bind GAL4, thus allowing transgene expression (McGuire et al., 2004; Figure 1A). In comparison to GFP, which was distributed relatively evenly through the cell, DmHDAC4 was distributed in both the axons and cell bodies of Kenyon cells with robust staining in the axons and in cytoplasmic haloes surrounding the nuclei (Figure 1B). Punctate foci were observed in a subset of nuclei as previously observed (Fitzsimons et al., 2013). Human HDAC4 also localized strongly to the axons, however, there was a much lower presence of hHDAC4 in nuclei, which is likely due to the nuclear export sequence in hHDAC4 that appears absent in DmHDAC4. The cytoplasmically-restricted L175A mutant was completely absent from nuclei, as expected. In contrast, neuronal nuclei of brains expressing 3SA contained numerous punctate foci, indicating nuclear retention, although it was not completely excluded from the cytoplasm as significant staining was also observed in the axons.

### Increased Nuclear HDAC4 Impairs Neuronal Development

**FIGURE 2:**
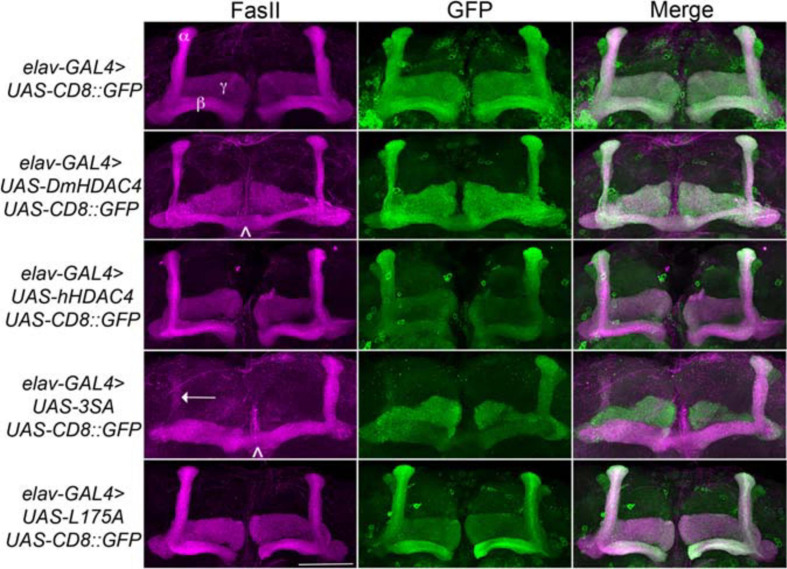
Nuclear accumulation of HDAC4 disrupts mushroom body development. Immunohistochemistry with anti-FasII and anti-GFP antibodies on whole mount brains expressing FLAG-tagged *HDAC4* variants and *CD8::GFP* as a counterstain to visualize all the lobes. All images are frontal confocal projections through the mushroom body region of the brain. All genotypes were generated by crossing *elav-GAL4; UAS-CD8::GFP* females to males carrying each *UAS-HDAC4* transgene and to the *w(CS10)* control. Brains displaying representative phenotypes are shown. Scale bar = 50 μm. α, β, and γ lobes of the mushroom body are labeled in white. Thin and fused β lobes (^∧^) are evident in a brain expressing *DmHDAC4*. A brain expressing *hHDAC4* appears normal. Missing α lobe (arrow) and fused β lobes (^∧^) are evident in a brain expressing *3SA*. A brain expressing *L175A* appears normal.

We next assessed the impact of increasing either nuclear or cytoplasmic HDAC4 on mushroom body development as we had previously observed that overexpression of wild-type *DmHDAC4* impairs axon branching, elongation and termination (unpublished data). The mushroom body is comprised of three classes of intrinsic neurons, the α/β, α′/β′ and γ Kenyon cells. The axons of the three Kenyon cell subtypes are bundled together to form a ventrally projecting peduncle, which then splits to form lobes. The α/β and α′/β′ axons both bifurcate to form the vertical α and α′ lobes and the medial β and β′ lobes, while the γ neuron axons form a single medial lobe. The integrity of the mushroom body can be assessed by *via* visualization of the α, β and γ lobe marker FasII (Freymuth and Fitzsimons, 2017; Figure 2). *elav-GAL4* driven pan-neuronal expression of wild-type *DmHDAC4* in post-mitotic neurons disrupted normal development in 95% of brains, manifesting as impairments in α/β lobe elongation and β-lobe fusion, which occurs when axons erroneously cross the midline. Defects resulting from expression of *hHDAC4* were significantly less pronounced than *DmHDAC4*, with 48% penetrance and the majority of the defects were thinner lobes (Table 1). Expression of *3SA* severely disrupted development with all brains displaying structural abnormalities, whereas in contrast, 85% of brains expressing cytoplasmic *L175A* appeared wild-type.

**TABLE 1:** Frequency of mushroom body phenotypes resulting from expression of *HDAC4* mutants.

**Genotype**	***elav* > *w(CS10)***	***elav* > *DmHDAC4***	***elav* > *hHDAC4***	***elav* > *3SA***	***elav* > *L175A***
*n*	23	21	23	31	21
No defects	100%	5%	52%	0%	85%
Thin α or β lobes	0%	19%	30%	19%	10%
Fused β lobes	0%	14%	9%	3%	5%
Thin α or β lobes and fused β lobes	0%	52%	4%	45%	0%
Absent α or β lobe	0%	10%	4%	23%	0%
Multiple absent lobes	0%	0%	0%	6%	0%

*The percentage of brains displaying each phenotype was calculated from the total number of brains analyzed for each genotype (*n*). Statistical analysis was performed with Fisher’s Exact Test. Expression of *DmHDAC4* resulted in significantly more abnormal brains than *hHDAC4* (*p* = 0.0007), as did *3SA* (*p* < 0.00001). *3SA* expression disrupted development to a significantly greater degree than *L175A* (*p* < 0.00001), which was not significantly different to the *w(CS10)* control (*p* = 0.104).*

**FIGURE 3:**
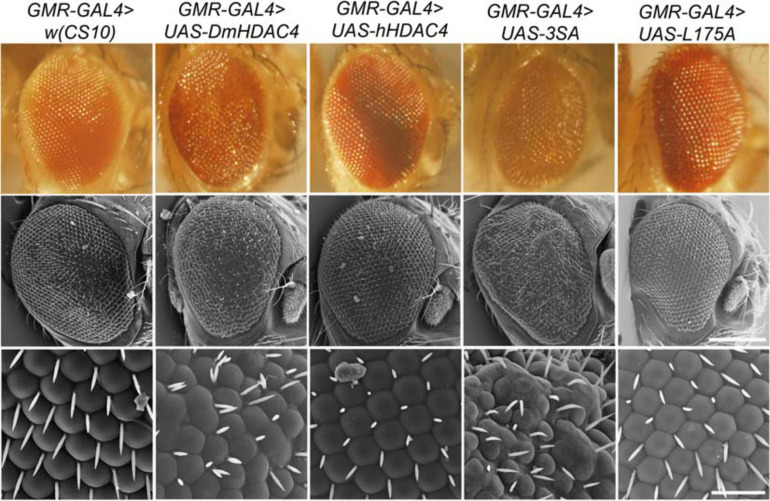
Nuclear accumulation of HDAC4 disrupts eye development. Stereomicrographs and scanning electron micrographs (SEM) of *Drosophila* eyes expressing FLAG-tagged *HDAC4* variants. The indicated genotypes were generated by crossing *GMR-GAL4* females to males carrying each *UAS-HDAC4* transgene and to the *w(CS10)* control. Top panel: Stereomicrographs, 110× magnification. Middle panel: SEM, 250× magnification. Scale bar = 200 μm. Lower Panel: SEM, 1,500× magnification. Scale bar = 30 μm.

We also examined whether nuclear accumulation of HDAC4 impacted eye development. The *Drosophila* eye is rich in neuronal photoreceptors and defects in its structure are readily observable. making it an excellent model for genetic analysis of neuronal pathways. We previously showed that expression of *DmHDAC4* in the post-mitotic eye *via* the glass multimer reporter (*GMR*) driver (Freeman, 1996) causes a disruption of the hexagonal ommatidia and bristles between them as well as a reduction in pigmentation, which was rescued *via* RNAi-mediated knockdown of *HDAC4* (Schwartz et al., 2016). Here we found that expression of *3SA* resulted in more severe deficits than *DmHDAC4* with reduced pigmentation, fused ommatidia and disorganized bristles, which was not observed on expression of wild-type *hHDAC4* nor *L175A* (Figure 3).

### Increased Nuclear HDAC4 Impairs Long-Term Memory

**FIGURE 4:**
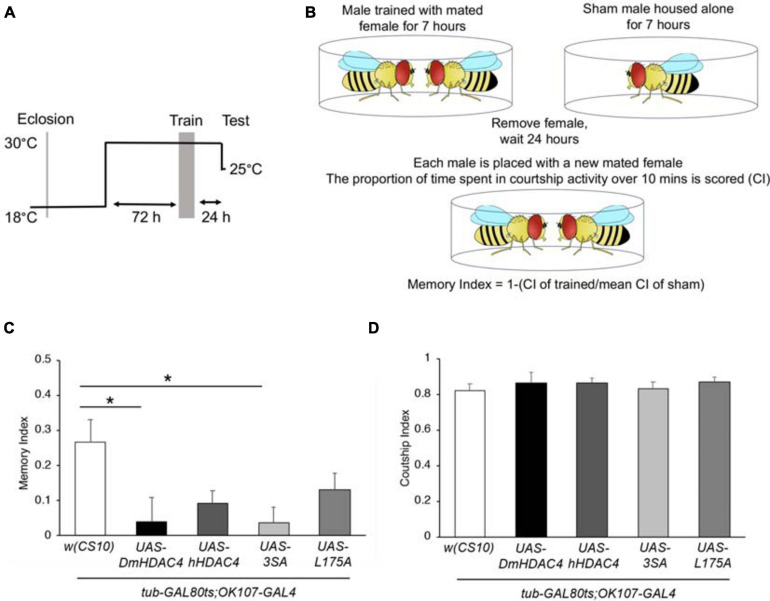
Nuclear accumulation of HDAC4 disrupts long-term memory. **(A)** Schematic diagram describing the protocol for induction of expression in the adult mushroom body. All genotypes were generated by crossing *OK107-GAL4; tubP-GAL80_ts_* females to males carrying the indicated UAS-transgene or to the *w(CS10)* control (CS). Flies were raised at 18°C until after eclosion, then placed at 30°C. They were trained 72 h later and remained at 30°C until 1 h prior to testing at which time they were equilibrated to the testing temperature of 25°C. **(B)** Schematic diagram describing the courtship suppression assay. **(C)** 24-h courtship LTM was significantly impaired by expression of *DmHDAC4* and *3SA* in the adult brain [ANOVA, F_(4,209)_ = 3.59, *p* < 0.007; *post-hoc* Tukey’s HSD, *p < 0.05]. **(D)** Courtship activity was not altered by expression of any of the *HDAC4* variants [ANOVA, *F*_(4,214)_ = 0.45, *p* = 0.772].

We next investigated whether nuclear HDAC4 was responsible for the long-term memory deficits we have previously observed on overexpression of *DmHDAC4* in the adult mushroom body (Fitzsimons et al., 2013). *OK107-GAL4*-driven expression was induced in the adult mushroom body (Figure 4A), and the courtship suppression assay was then used to evaluate memory. In this assay, a male fly’s courtship behavior is modified by his previous experience of rejection by a mated female, providing a quantitative assessment of associative memory (Figure 4B). Expression of *DmHDAC4* impaired LTM (Figure 4C) and while flies expressing *hHDAC4* displayed reduced LTM, this was not significant. Comparison of *hHDAC4* to *L175A* and *3SA* revealed that *3SA* impaired LTM to a significant level whereas *L175A* did not (Figure 4C). This was not due to a reduction in courtship behavior as naive flies of each genotype displayed normal courtship (Figure 4D). Taken together these data indicate that the nuclear accumulation of HDAC4 correlates with structural deficits in the developing mushroom body and eye, and impairs formation of LTM when expressed in the adult mushroom body.

### Characterization of Transcriptional Roles of HDAC4

**FIGURE 5:**
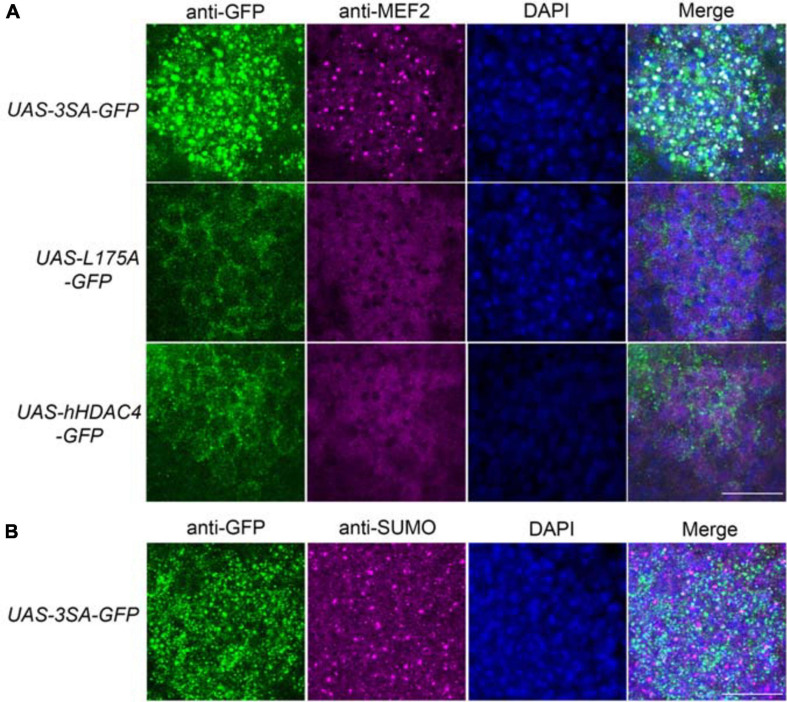
3SA distributes with MEF2 but not SUMO in nuclei. All genotypes were generated by crossing *tubP-GAL80ts; OK107-GAL4* females to males of the indicated genotype. Expression was induced in the adult brain by raising the flies at 18°C and placing at 30°C 72 h prior to dissection. Representative images from each genotype are shown. Z-stacks were captured of 0.25 μm slices, which were imaged at ×100 magnification. Whole-mount brains were subjected to immunohistochemistry with anti-MEF2 (magenta) and anti-GFP (green); or anti-SUMO (magenta) and anti-GFP (green). The number of overlapping puncta in each section (white in the merged image) were counted using ImageJ in each of ∼40 sections through the Kenyon cell layer. **(A)**. 3SA co-distributes with MEF2 in nuclei. 3SA colocalised with MEF2 (*n* = 7 brains, average number of puncta = 220 ± 9) whereas hHDAC4 (*n* = 7 brains, average number of puncta = 0) and L175A (*n* = 7 brains, average number of puncta = 0) did not. Scale bar = 10 μm. **(B)** 3SA does not co-distribute with SUMO in nuclei (*n* = 7 brains, average number of puncta = 0). Scale bar = 10 μm.

We previously demonstrated that increased nuclear abundance of DmHDAC4 results in the transcription factor MEF2 being sequestered into HDAC4-positive punctate nuclear foci (Fitzsimons et al., 2013). Similarly, expression of *3SA* also causes a redistribution of MEF2 from a relatively uniform distribution into numerous puncta in the nucleus, confirming that hHDAC4 also interacts with DmMEF2 when localized to the nucleus (Figure 5A). HDAC4 has been proposed to act as an E3 ligase, as it was demonstrated to enhance SUMOylation of MEF2 in mammalian cells (Gregoire and Yang, 2005; Zhao et al., 2005). We previously showed that *HDAC4* interacts genetically with the SUMOylation machinery and that the SUMO E3 ligase Ubc9 is required for LTM in *Drosophila*, but increased nuclear HDAC4 did not appear to alter global SUMOylation, nor facilitate SUMOylation of candidates MEF2, CREB, CaMKII or itself (Schwartz et al., 2016). This does not preclude that there could be changes in SUMOylation of specific unknown targets, and we hypothesized that HDAC4 may sequester SUMOylated proteins into the nuclear puncta observed in *3SA*-expressing nuclei. However, we saw no colocalization of SUMO with 3SA-positive foci (Figure 5B).

**FIGURE 6:**
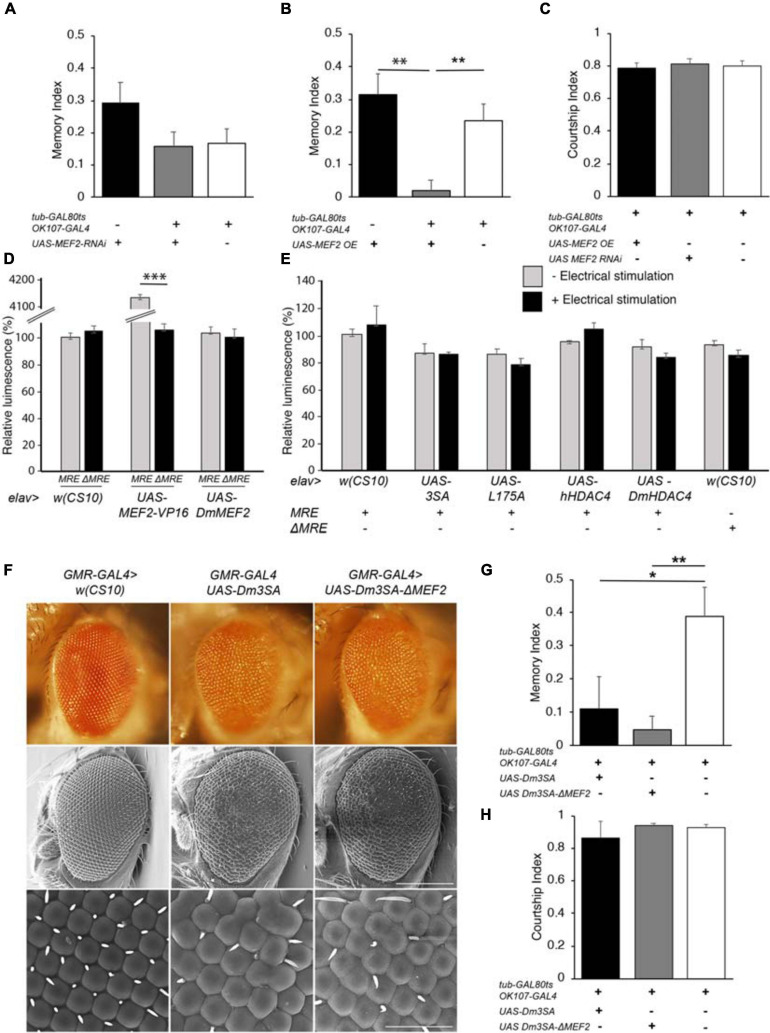
Functional analysis of the MEF2/HDAC4 interaction. **(A–C,G,H)** All genotypes were generated by crossing *OK107-GAL4; tub-GAL80_ts_* females to males of the indicated genotype. Expression was induced in the adult brain of progeny by raising the flies at 18°C until after eclosion, then placing at 30°C for 72 h. The courtship suppression assay was carried out as per Figure 4B. **(A)** *MEF2* knockdown in Kenyon cells does not alter LTM [ANOVA, *F*_(2,158)_ = 2.06, *p* = 0.13]. **(B)** Overexpression of *MEF2* in Kenyon cells impairs LTM [ANOVA, *F*_(2,156)_ = 10.91, *p* < 0.0001; *post-hoc* Tukey’s HSD, *p < 0.01]. **(C)** Courtship activity was not altered by knockdown or overexpression of *MEF2* [ANOVA, *F*_(2,174)_ = 1.62, *p* = 0.201]. **(D)** *elav-GAL4* driven expression of *MEF2-VP16* activated expression of luciferase in *MRE-luc* but not Δ*MRE-luc* brains [ANOVA, *F*_(5,18)_ = 1645, *p* < 0.000001; *post-hoc* Tukey’s HSD, *p < 0.000001]. Expression of *DmMEF2* did not increase luciferase activity above background levels in *MRE-luc* brains. **(E)** Electrical stimulation did not increase luciferase above background levels [*w(CS10*) control ± electrical stimulation, *t*-test *t*_(5)_ = 0.691, *p* = 0.520] and there was no alteration in luciferase activity on expression of wild-type or mutant *HDAC4* [ANOVA *F*_(5,17)_ = 1.46, *p* = 0.253]. **(F)** Stereomicrographs and scanning electron micrographs of *Drosophila* eyes in which the indicated transgenes are expressed with the *GMR-GAL4* driver. Top panel: Stereomicrographs. Middle panel: SEM 250× magnification. Scale bar = 200 μm. Lower Panel: SEM, 1,500× magnification. Scale bar = 40 μm. **(G)** Mutation of the MEF2 binding site did not prevent 3SA-induced impairment of LTM as compared to the control group [ANOVA, *F*_(2,29)_ = 5.53, *p* = 0.009; *post-hoc* Tukey’s HSD, *p < 0.05, **p < 0.01]. H. Courtship activity was unaffected by expression of *Dm3SA* or *Dm3SAΔMEF2* [ANOVA, *F*_(2,29)_ = 0.85, *p* = 0.438].

*DmHDAC4* and *DmMEF2* have been shown to interact genetically in a rough eye screen, indicating they act in the same molecular pathway during eye development (Schwartz et al., 2016), and MEF2 is required for normal mushroom body development in *Drosophila* (Crittenden et al., 2018). Together these data suggest the possibility that repression of MEF2-induced transcription may be a mechanism through which HDAC4 impairs memory and brain development in *Drosophila*. In this case, then reduction of *MEF2* expression should be expected to impair LTM, however, RNAi knockdown of *MEF2* had no significant impact on LTM (Figure 6A). Interestingly, overexpression of *DmMEF2* did impair LTM (Figure 6B), suggesting it is acting as a memory repressor. Neither knockdown nor overexpression of *DmMEF2* altered courtship activity itself (Figure 6C). Knockdown and overexpression of *DmMEF2* was verified *via* RT-qPCR and immunohistochemistry, respectively (Supplementary Figure 1).

We further investigated the interaction between HDAC4 and MEF2 *in vivo* with flies carrying a MEF2-response element (MRE) fused to luciferase. We confirmed that this cassette was functional *in vivo* by co-expression of a constitutively active *MEF2* transgene which consists of the MEF2 DNA binding domain fused to the VP16 activation domain (Chawla et al., 2003). When driven by *elav-GAL4*, MEF2-VP16 activated expression of luciferase in the adult brain, whereas no induction was observed in flies carrying Δ*MRE-luc*, confirming that the system was appropriately responsive to MEF2 *in vivo* (Figure 6D). Despite this, there was no significant difference in basal luciferase activity between *MRE-luc* and Δ*MRE-luc* flies, suggesting minimal endogenous MEF2 activity in the wild-type brain under basal conditions, and furthermore luciferase activity was not induced on overexpression of *DmMEF2*. Since MEF2 is activated by synaptic activity in mammals (Flavell et al., 2006), we induced neuronal depolarization *via* electrical stimulation of the brain with standard parameters used for olfactory conditioning (Qiu and Davis, 1993). However, this also resulted in no significant increase in luciferase activity above background levels (Figure 6E). Following our finding that overexpression of *MEF2* impaired memory, we investigated whether nuclear HDAC4 could possibly facilitate an increase in MEF2 activity, however, co-expression of wild-type or mutant *HDAC4* had no significant effect on luciferase activity in the absence of electrical stimulation, and there was no significant change in luciferase activity for any transgene when electrical stimulation was applied (Figure 6E).

To further examine the nature of a relationship between HDAC4 and MEF2, we generated flies carrying *3SA* with a mutated MEF2 binding site to determine whether the 3SA phenotype was dependent on MEF2 binding. We first generated the corresponding *3SA* mutation in *DmHDAC4* to confirm conserved functionality between *Drosophila* and human HDAC4, and observed that like human *3SA*, *Dm3SA* expression in the eye resulted in loss of pigmentation, fusion of ommatidia and missing or misplaced bristles (Figure 6F). Mutation of the MEF2 binding site (*Dm3SA-ΔMEF2*) resulted in a phenotype indistinguishable from *Dm3SA* alone, indicating that an interaction with MEF2 is not required for the nuclear HDAC4-induced phenotype. Similarly, when expressed in the adult mushroom body, Dm3SA-ΔMEF2 retained the ability to impair long-term memory (Figure 6G) and we again confirmed that this was not due to an alteration courtship behavior (Figure 6H). Dm3SA-ΔMEF2 displayed a significantly reduced co-localization with DmMEF2 in nuclear puncta (Supplementary Figure 2) confirming that the HDAC4-MEF2 interaction was disrupted in this mutant.

When examined for the effects on axon elongation and termination in the mushroom body, expression of *Dm3SA* resulted in a similar phenotype to *h3SA* with missing and fused lobes. However, this phenotype was significantly reduced in brains expressing *Dm3SA-*Δ*MEF2* (Table 2), which suggested that MEF2 binding is crucial for the 3SA-induced developmental defects in the mushroom body. Co-expression of *Dm3SA* and *MEF2* RNAi also significantly reduced the defects, providing further evidence that 3SA acts through MEF2.

**TABLE 2:** Frequency of mushroom body phenotypes resulting from expression of *Dm3SA* in the presence and absence of the MEF2 binding site.

**Genotype**	***elav* > *w(CS10)***	***elav* > *Dm3SA***	***elav* > *Dm3SA-*Δ*MEF2***	***elav* > *Dm3SA, MEF2 RNAi***	***Elav > MEF2 RNAi***
*N*	20	34	34	33	20
No defects	100%	9%	62%	42%	90%
Thin α or β lobes	0%	6%	6%	9%	0%
Fused β lobes	0%	65%	9%	21%	0%
Thin α or β lobes and fused β lobes	0%	11%	0%	21%	0%
Absent α or β lobe	0%	9%	23%	7%	10%

*The percentage of brains displaying each phenotype was calculated from the total number of brains analyzed for each genotype (*n*). Statistical analysis was performed with Fisher’s Exact Test. Expression of *Dm3SA* resulted in significantly more abnormal brains than *Dm3SA-*Δ*MEF2* (*p* < 0.00001), and *Dm3SA* coexpressed with *MEF2* RNAi (*p* = 0.002). There was no significant difference between *Dm3SA-*Δ*MEF2 and 3SA, MEF2* RNAi (*p* = 0.145).*

**FIGURE 7:**
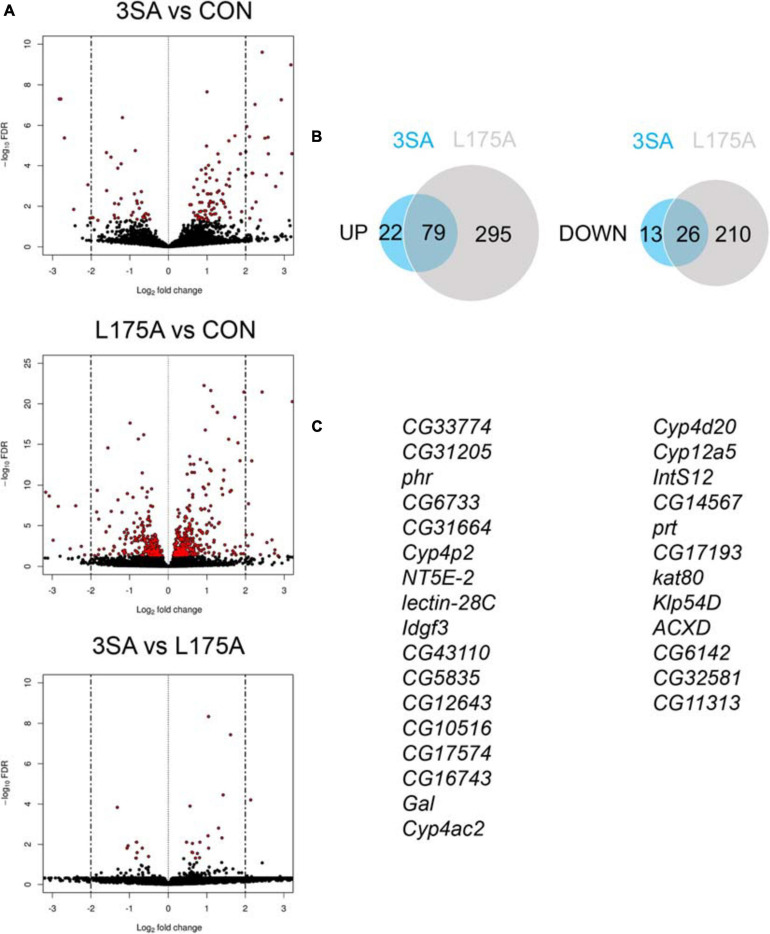
Transcriptional changes resulting from expression of 3SA and L175A. **(A)** Genotypes were generated by crossing *tubP-GAL80ts; OK107-GAL4* females to *UAS-FLAG-3SA*, *UAS-FLAG-L175A* or *w(CS10)* (indicate as CON) males. Expression was induced in the adult brain by raising the flies at 18°C and placing at 30°C 72 h prior to dissection. Volcano plots indicate differential expression between treatment groups. The horizontal axes indicate the Log2-fold change in expression and the vertical axis indicates the negative Log10 of the false discovery rate. Genes with significant differential expression (i.e., FDR < 0.05) are highlighted in red. Expression of *L175A* resulted in a higher number of differentially expressed genes than *3SA* (2-sample test for equality of proportions (X-squared = 296.89, df = 1, *p*-value < 0.01). **(B)** Venn diagrams indicating the number of differentially regulated genes common to both control vs. *L175A* and control vs. *3SA* data sets **(C)** List of genes significantly altered in expression by 3SA but not L175A. Only 29 genes were common to both data sets.

Given that we could not detect transcriptional activity of MEF2, we examined whether expression of *3SA* resulted in any changes in gene expression *via* RNA-seq. Previous RNA-seq on heads of flies overexpressing *DmHDAC4* in the adult fly brain revealed few significant transcriptional changes and we reasoned that this may be due to a dilution effect, given that wild-type DmHDAC4 is nuclear in only a subset of cells. We therefore performed RNA-seq on heads of flies expressing *3SA* in the adult brain. Developmental effects were avoided by induction of expression in adult brains with GAL80ts (Figure 1A). Very few transcriptional changes were observed (Figure 7A and Supplementary Table 1), leading us to conclude that even when sequestered in all neuronal nuclei, HDAC4 elicits only small changes in transcription in the fly brain. Surprisingly, there were significantly more differentially expressed genes in the *L175A* group versus *3SA* and the majority of genes that were differentially regulated by *3SA* were also altered by *L175A* (Figure 7B). Given that L175A appeared entirely cytoplasmic but 3SA was not completely restricted to the nucleus with some distribution in the cytoplasm (Figure 1B), it is a logical assumption that the change in expression of these genes was mediated by cytoplasmic HDAC4. A direct comparison of *3SA* and *L175A* revealed only 29 genes were significantly differentially expressed with a False Discovery Rate < 0.05 (Figure 7C and Supplementary Table 1) and only four of these genes displayed more than a Log2-fold change in expression. Gene ontology (GO) analysis using the differentially expressed list from the *L175A* and *3SA* comparison identified enrichment of only four molecular functions: monooxygenase activity, paired donor oxidoreductase activity, heme binding and iron ion binding; and the source of these GO terms was only four genes that encode cytochrome P450 superfamily proteins (Supplementary Table 1).

## Discussion

Here we demonstrate that the neurological impairments resulting from increased *HDAC4* expression correlate with increased abundance of nuclear HDAC4. Expression of wild-type and cytoplasmic *hHDAC4* resulted in minimal phenotypic changes whereas nuclear accumulation resulted in severe defects in mushroom body and eye development, as well as disruption of long-term memory. These data establish that *Drosophila* is an informative model for investigation of the molecular mechanisms that underlie disorders associated with nuclear accumulation of HDAC4.

Expression of *3SA* in nuclei of post-mitotic neurons caused deficits in elongation and termination of Kenyon cell axons. In contrast, in the mouse forebrain, expression of a nuclear-restricted truncated mutant of *HDAC4* did not alter gross architecture of the hippocampus nor neuronal survival as evidenced by number of dentate granule cells and densities of their synapses (Sando et al., 2012). In addition, brain-specific knockout of *HDAC4* in the mouse has no major impact on normal morphology or survival (Price et al., 2013) and nor does double *HDAC4/HDAC5* knockout (Zhu et al., 2019) suggesting HDAC4 does not play a role in development of an otherwise normal rodent brain. However, vertebrates have four Class IIa HDACs while *HDAC4* is the sole Class IIa HDAC in *Drosophila*, which avoids effects of redundancy and allows the basic functions of Class IIa HDACs to be uncovered. There are some instances, however, in which nuclear accumulation of HDAC4 impacts brain development in vertebrates when associated with existing disease. Cyclin dependent kinase-like 5 (CDLK5) disorder is a neurodevelopmental disorder associated with intellectual disability, and *CDLK5* mutant mice display deficits in neuronal survival and maturation as well as impaired hippocampal-dependent memory. HDAC4 is a phosphorylation target of CDLK5 and in its absence, nuclear entry of unphosphorylated HDAC4 is associated with reduced histone H3 acetylation and modest repression of *MEF2*. Treatment with an HDAC4 inhibitor restores memory and reverses defects in neuronal maturation and death, which is highly suggestive that the deficits associated with *CDLK5* disorder are a result of nuclear accumulation of HDAC4 (Trazzi et al., 2016). The expression of *3SA* in the adult mushroom body also abolished the formation of long-term memory, which is consistent with findings that a truncated mutant of human HDAC4 that accumulates in nuclei impairs memory (Sando et al., 2012). It is notable that this mutant also lacks a deacetylase domain, suggesting a deacetylase-independent mechanism. Taken together, it is evident that when overabundant in the nucleus, HDAC4 disrupts neuronal morphogenesis and memory in both vertebrates and invertebrates. We previously identified that *HDAC4* interacts genetically with several cytoskeletal regulators including *Moesin* and *Ankyrin2*, although it does not regulate their expression (Schwartz et al., 2016). While the nature of the interaction is yet to be determined, we also showed that knockdown of *Moesin* resulted in similar mushroom body deficits and impaired long-term memory (Freymuth and Fitzsimons, 2017) as does knockdown of *Ankyrin2* (unpublished data). Together these data suggest that nuclear HDAC4 may be an upstream regulator of molecular pathways that regulate rearrangement of the actin cytoskeleton.

The most obvious mechanism through which HDAC4 would alter neuronal development and memory would be modulation of gene expression, however, we previously found that overexpression of *DmHDAC4* was associated with only minor transcriptional changes (Schwartz et al., 2016). As the neuronal distribution of HDAC4 is largely cytoplasmic we reasoned that any significant changes in neuronal populations may have been diluted and could be uncovered by forcing HDAC4 to accumulate in nuclei of all neurons. However, again we saw minimal changes resulting from forced nuclear accumulation *via* expression of *3SA* and surprisingly the majority of differential gene expression was a result of cytoplasmic accumulation of HDAC4. Comparison with our previous RNA-seq data in which *DmHDAC4* was overexpressed (Schwartz et al., 2016) revealed that of the 26 genes differentially expressed in that study (not including *w* and *DmHDAC4*), 14 were also differentially expressed following expression of *L175A*, approximately 54% of the total. These data indicate conservation of function between of HDAC4 between humans and *Drosophila* and surprisingly, that the changes elicited by DmHDAC4 are very likely through its cytoplasmic presence. How HDAC4 facilitates alterations in gene expression from the cytoplasm is unknown, but it could be through sequestration of transcriptional regulators in the cytoplasm, which has previously been demonstrated for the transcription factor ATF4, whereby increased cytoplasmic HDAC4 redistributed ATF4 from the nucleus to the cytoplasm (Zhang et al., 2014). It is clear that in the data we present here, these transcriptional changes resulting from increased cytoplasmic HDAC4 are not associated with any observed neurological phenotypes. However, knockdown of *HDAC4* impairs LTM in *Drosophila* (Fitzsimons et al., 2013) as does brain-specific knockout of *HDAC4* (Kim et al., 2012) and *HDAC4/5* (Zhu et al., 2019) in mice, indicating it plays an essential role in normal memory formation. It therefore warrants further investigation as to whether the same genes and/or pathways are altered in the opposite direction upon reduction of HDAC4, which would provide insight into whether loss of cytoplasmic HDAC4 contributes to the memory deficits resulting from a reduction in *HDAC4* expression.

We observed that both human and *Drosophila* HDAC4 interact with MEF2, and when abundant in the nucleus they sequester MEF2 into punctate nuclear foci in Kenyon cells. Given that Kenyon cells are critical to formation of memory, and that HDAC4 is well established as a repressor of MEF2, it was a logical assumption that HDAC4 may impair memory by repressing MEF2. However, the lack of transcriptional changes does not support this and moreover, knockdown of *MEF2* in the adult brain had no impact on LTM. On the contrary, overexpression of *MEF2* significantly impaired LTM. This is consistent with evidence that MEF2 is a memory repressor in mice; inhibitory phosphorylation of MEF2 in the murine hippocampus or nucleus accumbens is associated with the formation of normal spatial and fear memories and overexpression of MEF2 in the dentate gyrus and amygdala blocks both spatial memory and fear memory, respectively (Cole et al., 2012). Together these data provide evidence that the mechanism through which nuclear sequestration of HDAC4 impairs memory in *Drosophila* is likely not *via* MEF2 repression. We similarly demonstrated that in the developing eye, 3SA does not require MEF2 binding to impair development. In contrast, in the developing mushroom body, mutation of the MEF2 binding site prevented most of the 3SA-induced defects, as did knockdown of *MEF2*, which implies that 3SA has a differential requirement for MEF2 in the developing versus the adult mushroom body.

Here we have shown that the irreversible accumulation of HDAC4 in the nucleus is associated with neurological dysfunction, and both we and others have observed that when overexpressed, HDAC4 aggregates into punctate foci within nuclei (Miska et al., 1999; Borghi et al., 2001; McKinsey et al., 2001; Fischle et al., 2002; Fitzsimons et al., 2013). The propensity of HDAC4 to aggregate stems from its glutamine-rich N-terminus which forms an alpha helix that can assemble into tetramers and further oligomerize. Interestingly, HDAC4-positive nuclear inclusions have been associated with pathogenesis of neurological disorders including Lewy bodies in Parkinson’s disease and intra-nuclear inclusions in intra-nuclear inclusion disease (Takahashi-Fujigasaki and Fujigasaki, 2006). These inclusions also contain SUMO (Takahashi-Fujigasaki et al., 2006), which was of interest given our previous finding that *HDAC4* interacts in the same molecular pathway as several genes involved in SUMOylation (Schwartz et al., 2016) however, SUMO did not appear to be a component of the aggregates in *Drosophila* Kenyon cells. The possibility remains that SUMOylation of other nuclear proteins may be impacted by HDAC4, however, we did not see any alteration in overall pattern of SUMO localization on expression of *3SA*. Given that the deficits in neuronal morphogenesis and memory are facilitated by nuclear HDAC4 which has formed into these aggregates, the identity of other aggregate components and the role of HDAC4 aggregation in neuronal function warrant further investigation.

## Materials and Methods

### Fly Strains

All flies were raised on standard medium on a 12-h light/dark cycle and maintained at a temperature of 25°C unless otherwise indicated. *w[^∗^]; P{w[+mW.hs] = GawB}OK107 ey[OK107]/In(4)ci[D], ci[D] pan[ciD] sv[spa-pol]* (*OK107-GAL4*), *w[^∗^]; P{w[+mC] = GAL4-ninaE.GMR}12* (*GMR-GAL4*), *P{w[+mW.hs] = GawB}elav[c155]* (*elav-GAL4*) and *y1w^∗^; P{UAS-mCD8::GFP.L}LL5* (*UAS-CD8::GFP*) were obtained from the Bloomington *Drosophila* Stock Center. *w^1118^; P{attP,y[+],w[3′] CG42734* (*UAS-MEF2* RNAi) was obtained from the Vienna *Drosophila* Resource Center. *w^∗^; P{w+mC = tubP-GAL80ts}10; TM2/TM6B, Tb1* (*tubP-GAL80_ts_*) and *w(CS10)* strains were kindly provided by R. Davis (The Scripps Research Institute, Jupiter, FL). FLAG-tagged *Drosophila* and human *HDAC4* and the human *3SA* and *L175A* mutant lines have been described previously (Fitzsimons et al., 2013; Schwartz et al., 2016). GFP-tagged *HDAC4* variants were generated by amplification with NotI linkers and insertion of each variant into pUAST-GFP which contained a unique NotI site downstream of GFP to create in frame N-terminal fusions of GFP. *MRE-luc* was generated by annealing the oligos containing 3x consensus MEF2 binding sites (Andres et al., 1995). 3xMREfor 5′ ctagcgatatctgttactaaaaatagaatgttactaaaaatagaatgttactaaaaatagaaa 3′ and 3xMRErev 5′ gatctttc tatttttagtaacattctatttttagtaacattctatttttagtaacagat 3′, digesting with NheI/BglII and inserting into NheI/BglII digested pUAST to create pattBMRE. The minimal hsp70 promoter was PCR amplified from pUASTattB with the following primers hsp70 for 5′ atctagatctgagcgccggagtataaatag 3′ and hsp70rev 5′ atcgctcgagggatcccaattccctattcagagttc3′ with BglII and XhoI linkers and cloned into BglII/XhoI of pattB-MRE. pMIR-report (Ambion) was digested with BamHI/SpeI to release the luciferase gene which was cloned into BamHI and XbaI of pattB-MRE to create pattB-MRE-luc. *DMRE-luc* was similarly constructed with the oligos 3xDMREfor 5′ ctagcgatatctgttactaagggtagaatgttactaagggtagaatgt tactaagggtaga 3′ and 3xDMRErev 5′gatctttc tacccttagtaacattcta cccttagtaacattctacccttagtaacagatatc 3′. *Drosophila HDAC4* was synthesized by Genscript (New Jersey, United States) (nucleotides 461–4216 of NCBI reference sequence NM_132640 with a C-terminal 6x Myc tag) and mutagenesis was performed on this construct to generate the *3SA* and *DMEF2* mutants. The 3SA amino acid substitutions are S239A, S573A, and S748A and DMEF2 amino acid substitutions within the MEF2 binding site are K165A L168A and I172A. *DmMEF2* was synthesized by Genscript (nucleotides 1,057–2,601 of NCBI reference sequence NM_057670.5 with a C-terminal 3x HA tag). *DmMEF2* was similarly subcloned with an N-terminal 6xMyc tag, and the *HDAC4* and *MEF2* constructs were cloned into the pUASTattB plasmid for germline transformation of *Drosophila*. Transgenic flies were generated by Genetivision (Houston, TX) using the P2 docking site at (3L)68A4 for the *HDAC4* constructs, the VK22 docking site at (2R)57F5 for *MEF2-HA*, the (3L)68E1 site for *Myc-MEF2* and the VK37 docking site at (2L) 22A3 for *MRE-luc* and *DMRE-luc*. All strains were outcrossed for a minimum for five generations to *w(CS10)* flies. Homozygous lines harboring the appropriate GAL4 driver and *tubP-GAL80ts* were generated by standard genetic crosses.

### Scanning Electron Microscopy

Flies were anesthetized using FlyNap (Carolina Biologicals) and added to primary modified Karnovsky’s fixative (3% glutaraldehyde, 2% formaldehyde in 0.1 M phosphate buffer, pH 7.2) with Triton X-100 and vacuum infiltrated until soaked through. The flies were then placed in fresh fixative and incubated at RT for at least 8 h. Three 10-min phosphate buffer (0.1 M, pH 7.2) washes were carried out followed by dehydration using a graded ethanol series for ten to 15 min at each step (25, 50, 75, 95, and 100%) and a final 1-h wash in 100% ethanol was carried out. Following the ethanol dehydration, the samples were critical point dried using CO_2_ and 100% ethanol (Polaron E3000 series II drying apparatus). The samples were then mounted onto aluminum stubs and sputter coated with gold (Baltex SCD 050 sputter coater) and imaged in the FEI Quanta 200 Environmental Scanning Electron Microscope at an accelerating voltage of 20 kV.

### Behavioral Analyses

The repeat training courtship suppression assay (Keleman et al., 2007; Ejima and Griffith, 2011) was used to assess 24-h long-term memory. Male flies learn that they have been previously rejected by a female and thus when tested with a new female, they display a decrease in courtship behavior compared to naïve males. The detailed methodology has been described previously (Fitzsimons and Scott, 2011; Freymuth and Fitzsimons, 2017). Briefly, single virgin males of each genotype were placed into individual training chambers. A freshly mated wild-type female was placed into half of the chambers (trained group), and then other half of the male group was housed alone (sham group). Flies were incubated in the training chamber for 7 h, during which time multiple bouts of courting were observed in the trained group. The female fly was then aspirated from the training chamber and the males were left in their chambers for the 24 h prior to testing.

Each trained or naïve male fly was then placed into a testing chamber containing a mated wild type female and was scored for the time spent performing stereotypic courtship behaviors over the 10-min period. A courtship index (CI) was calculated as the proportion of the 10-min period spent in courtship behavior. A mean CI for each group was determined, and from this a memory index (MI) was calculated by the following equation: MI = 1-(CI of each trained fly/mean CI of sham group) (*n* ≥ 16/group). The MI was measured on a scale of 0 to 1, a score of 0 indicating memory was no different than untrained naive controls. In all experiments, the scorer was blind to the genotype of the flies.

### Immunohistochemistry

Whole flies were fixed in PFAT/DMSO (4% paraformaldehyde in 1X PBS +0.1% Triton X-100+5% DMSO) for 1 h then microdissected in 1XPBS. Brains were post-fixed in PFAT/DMSO for 20 min and blocked in immunobuffer (5% normal goat serum in 1XPBS+0.5% Triton X-100) for 2 h prior to incubation with primary antibody of mouse anti-FasII (1:200); mouse anti-SUMO-2 (1:20), rabbit anti-GFP (Abcam ab290 1:1,000), rat anti-HA (3F10 Sigma Aldrich 1:500) mouse anti-Myc (1:100) or rabbit anti-MEF2 (gift from Bruce Paterson, National Cancer Institute, Bethesda, 1:500). They were then incubated with secondary antibody (goat anti-mouse Alexa 488 or 555, goat anti-rabbit Alexa 488 or 555, or goat anti-rat Alexa 555, Sigma Aldrich, 1:500) and mounted with Antifade. The monoclonal antibodies anti-Fasciclin II (1D4, developed by G. Goodman), anti-Myc (9E 10, developed by J. M. Bishop) and anti-SUMO-2 (A82, developed by M. Matunis) were obtained from the Developmental Studies Hybridoma Bank developed under the auspices of the NICHD and maintained by The University of Iowa, Department of Biology, Iowa City, IA 52242. For confocal microscopy, images were captured using a Leica TCS SP5 DM6000B Confocal Microscope and images were processed with Leica Application Suite Advanced Fluorescence (LAS AF) software. To quantify puncta foci in Kenyon cells in which 3SA co-localized with MEF2 or SUMO, Z-stacks were captured of ∼40 0.25 μm slices through the same region of the Kenyon cell layer starting at the posterior of the calyx, and were imaged at x100 magnification. The number of overlapping puncta in each section (white in the merged image) were counted using ImageJ in each of through the Kenyon cell layer. Foci were only counted if they were visible in both single and merged channels, and when puncta were present in adjacent slices they were only counted once.

### RT-qPCR

*elav-GAL4* females were crossed to *UAS-MEF2 RNAi* males to generate progeny in which *MEF2* was knocked down in all neurons; and progeny of *elav-GAL4* crossed to *w(CS10)* served as controls. Total RNA was extracted from *Drosophila* heads from three independent crosses for each genotype with the RNeasy Mini kit (Qiagen) according to the manufacturer’s instructions. cDNA was synthesized from 1 μg of total RNA with Transcriptor (Roche) as per the manufacturer’s instructions. RT-qPCR was conducted using SsoFast-EvaGreen (BioRad) reaction master on a Lightcycler II 480 instrument (Roche), following manufacturer’s instructions. The following primers were used: *Mef2*for 5′-GCCACATCACACCCACTCC-3′, *Mef2*rev 5′-GCTGGCCATAGCAGTCGTAG-3′, *EF1a48D*for *5*′*-*ACTTT GTTCGAATCCGTCGC-3′, *EF1a48D*rev 5′-TACGCTTGTCGA TACCACCG-3′. A fivefold dilution of cDNA from control flies was used as template to prepare a standard curve to confirm efficiency of the PCR reactions. Relative quantification was conducted using 2^–ΔΔCt^ method, normalizing to the housekeeping gene *Ef1α48D* (Livak and and Schmittgen, 2001).

### Luciferase Assays

*elav-GAL4; MRE-luc* and *elav-GAL4; ΔMRE-luc* females were crossed to males of the appropriate genotype. Luciferase assays were performed on the heads of 3–5 day old adults. Heads (10/sample) were homogenized in 20 μL Glo Lysis buffer (Promega), incubated at room temperature for 15 min, then centrifuged at 12,000 *g* for 10 min. 10 μL of each supernatant was applied per well for each genotype. Quadruplicate samples were analyzed for each genotype. 40 μL Bright Glo reagent was added to each well and then luminescence was measured on a PolarStarOMEGA luminometer (BMG Labtech) and normalized to the *w(CS10)* control luminescence. Flies were electrically stimulated by placing them in a modified 15 mL plastic tube lined with an electrified grid and exposed to 12 electric shocks (90 V, 1.5 s) over 1 min and harvested after 1.5 h to allow transcription and translation of luciferase.

### Transcriptome Analysis

Female *elav-GAL4; tubP-GAL80ts* flies were crossed to males individually carrying *UAS-hHDAC4-3SA* or *UAS-hHDAC4-L175A* as well as to *w(CS10)* to provide a control line with the same genetic background and regulatory constructs but no *UAS-HDAC4* construct. Four biological replicates (independent crosses) were generated per genotype. Flies were raised at 18°C then 3–5 day old adult flies were incubated at 30°C for 72 h to induce expression of *3SA* and *L175A* in all neurons. The flies were then snap frozen in liquid nitrogen and their heads separated by vortexing and sorted from bodies by sieving. RNA extracted from *Drosophila* heads (RNeasy mini kit, Qiagen) was quantified on a Denovix DS-11 spectrophotometer and assessed for quality on a Labchip GX Touch HT (PerkinElmer). Illumina HiSeq 150 bp paired-end sequencing was carried out by Novogene (Hong Kong). The sequencing yielded an average paired end read count of 36.9 million per sample replicate. Read mapping and read count analysis was performed by NextGen Bioinformatic Services (New Zealand). The raw read data was adaptor filtered and quality trimmed (phred > 10) using BBDuk (version 36.86; BBTools by B Bushnell^1^). Following quality trimming any reads shorter than 50 bp were removed to minimize the possibility of multiple mapping. The BBDuk parameters were: minlen = 50 qtrim = rl trimq = 10 ktrim = r k = 21 mink = 11. The data was observed to be high quality with over 99% of bases passing the filtering and quality trimming step. High quality reads were mapped to the *Drosophila* genome (release 92) downloaded from ENSEMBL using HISAT2 (version 2.05) in stranded mapping mode. The alignment rate to the genome was > 94% of reads for all sample replicates. RNA-seq reads from the *3SA* and *L175A* libraries were also mapped to human chromosome 2 (assembly version GRCh38) to verify the presence of the mutations in the samples from of these two treatment groups. Gene based read counts were generated from the alignment BAM files using HT-Seq (version 0.6.0) based on the “union” mode. The count files were processed in R (version 3.4.0) and differentially expressed genes were identified using the package DESeq2 (version 1.18.1) as described in the vignette (Love et al., 2014). Differentially expressed genes with an FDR < 0.05 (i.e., significantly altered) were uploaded to the Database for Analysis, Visualization and Integrated Discovery (DAVID, 6.8) (Huang Da et al., 2009) to identify functional groups highly enriched in a sample, as well as identifying biological pathways that are highly represented in a given group.

## Data Availability Statement

The NCBI bioproject number for this study is PRJNA664892 (https://www.ncbi.nlm.nih.gov/bioproject/PRJNA664892) and the raw sequencing data is available at the NCBI Short Read Archive (https://www.ncbi.nlm.nih.gov/sra) under accession numbers SRR12689988-SRR12689999.

## Author Contributions

HF, PM, and WT designed the study. HF directed the study and carried out the luciferase assays. PM carried out the majority of the experiments. WT performed light microscopy, SEM, and immunohistochemistry on the double mutant. DW performed the bioinformatic analyses. HF wrote the manuscript with assistance from PM and WT (Main et al., 2021). All authors contributed to the article and approved the submitted version.

## Conflict of Interest

The authors declare that the research was conducted in the absence of any commercial or financial relationships that could be construed as a potential conflict of interest.

## Footnotes

1 www.sourceforge.net/projects/bbmap/

## Supplementary Material

Supplementary Figure 1 Confirmation of MEF2 knockdown and overexpression. A. *elav-GAL4* females were crossed *UAS-MEF2 RNAi* males in order to knock down *MEF2* in all neurons. RNA was isolated from heads and RT-qPCR for *MEF2* was carried out with normalization to *Ef1α48D. MEF2* was significantly knocked down to approximately 40% of wild-type [*t*-test t_(4)_ = 3.99, *p* < 0.05]. B. *OK107-GAL4; tubP-GAL80_ts_* females were crossed to *UAS-Myc-MEF2* or *w(CS)10* males. Flies were raised at 18°C until after eclosion, then placed at 30°C 72 h prior to dissection to induce transgene expression. B. Whole-mount brains were processed for immunohistochemistry with anti-MEF2 (green) and anti-Myc (magenta). Optical sections (1 μm) through the Kenyon cells show that Myc-MEF2 co-localizes with endogenous MEF2 in Kenyon cell nuclei, whereas there is no Myc-MEF2 expression in the control. Scale bar = 50 μm.

Supplementary Figure 2 Mutation of the MEF2 binding site reduces binding of 3SA to MEF2. *OK107-GAL4; tub-GAL80_ts_* females were crossed to *UAS-Dm3SA* and *UAS-Dm3SA-ΔMEF2* males, and expression was induced in the brains of adult progeny by raising the flies at 18°C until after eclosion, then placing at 30°C for 72 h. A. Whole-mount brains were subjected to immunohistochemistry with anti-Myc (green) and anti-MEF2 (magenta). Z-stacks were captured of 0.25 μm slices, which were imaged at x100 magnification. Overlapping puncta appear white in the merged channel. B. The number of overlapping puncta in each section were counted using ImageJ in each of ∼40 sections through the Kenyon cell layer. Dm3SA-ΔMEF2 (*n* = 7 brains) displayed significantly reduced co-distribution with MEF2 in comparison to Dm3SA (*n* = 5 brains) [*t*-test *t*_(10)_ = 6.318, *p* < 0.0001]. Scale bar = 10 μm.

Supplementary Table 1 Differentially expressed genes between control and L175A; control and 3SA; and 3SA and L175A groups. Genes with a False Discovery Rate of < 0.05 are shown.
