# Supplementary figures and images for: Increased Abundance of Nuclear HDAC4 Impairs Neuronal Development and Long-Term Memory
Source: Front Mol Neurosci. 2021 Mar 30;14:616642. doi: 10.3389/fnmol.2021.616642 (PMC8042284; doi:10.3389/fnmol.2021.616642)

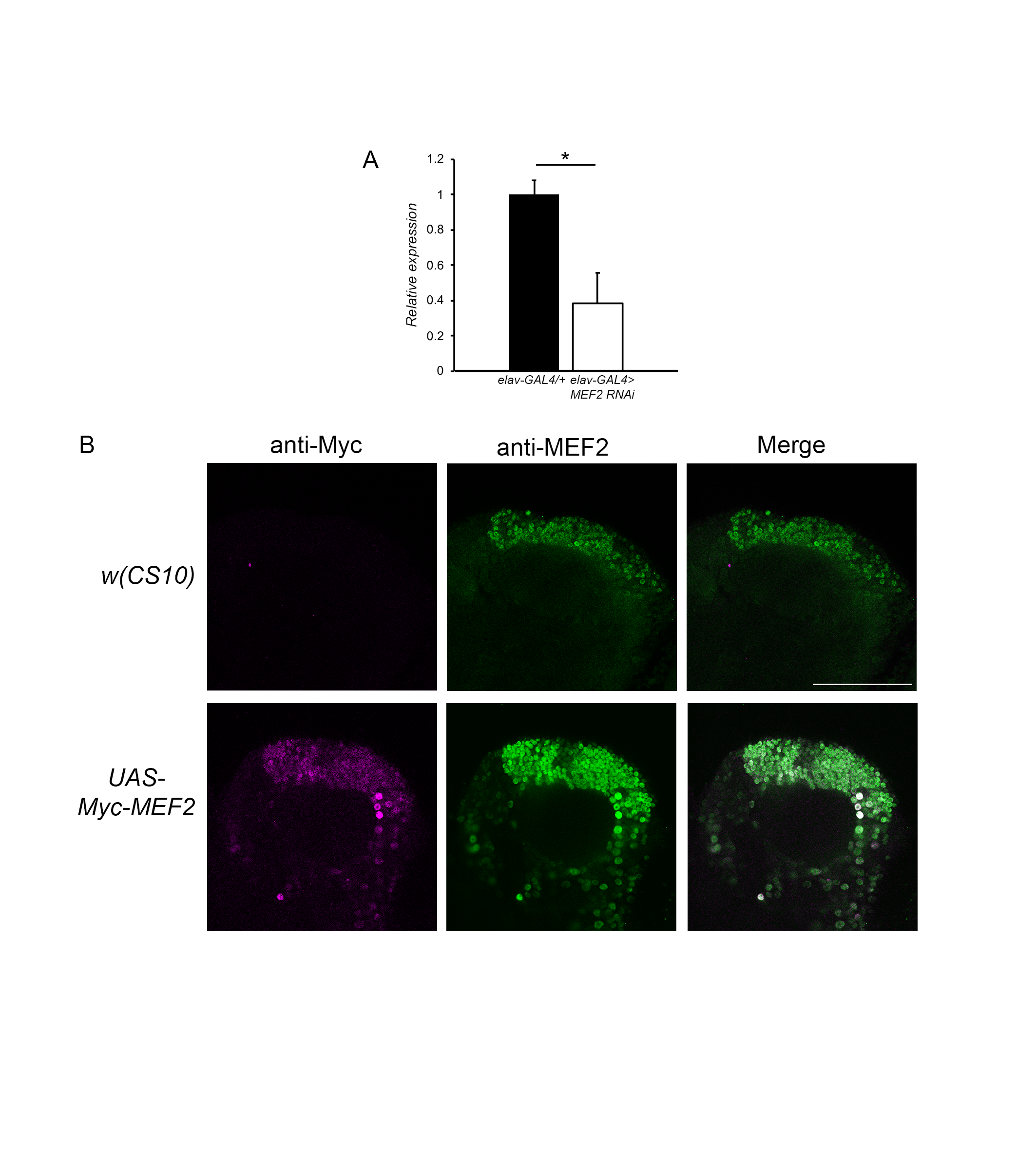

Supplement: Supplementary Figure 1 — Confirmation of MEF2 knockdown and overexpression. A. elav-GAL4 females were crossed UAS-MEF2 RNAi males in order to knock down MEF2 in all neurons. RNA was isolated from heads and RT-qPCR for MEF2 was carried out with normalization to Ef1α48D. MEF2 was significantly knocked down to approximately 40% of wild-type [t-test t(4) = 3.99, p < 0.05]. B. OK107-GAL4; tubP-GAL80ts females were crossed to UAS-Myc-MEF2 or w(CS)10 males. Flies were raised at 18°C until after eclosion, then placed at 30°C 72 h prior to dissection to induce transgene expression. B. Whole-mount brains were processed for immunohistochemistry with anti-MEF2 (green) and anti-Myc (magenta). Optical sections (1 μm) through the Kenyon cells show that Myc-MEF2 co-localizes with endogenous MEF2 in Kenyon cell nuclei, whereas there is no Myc-MEF2 expression in the control. Scale bar = 50 μm. [file Image_1.TIF]

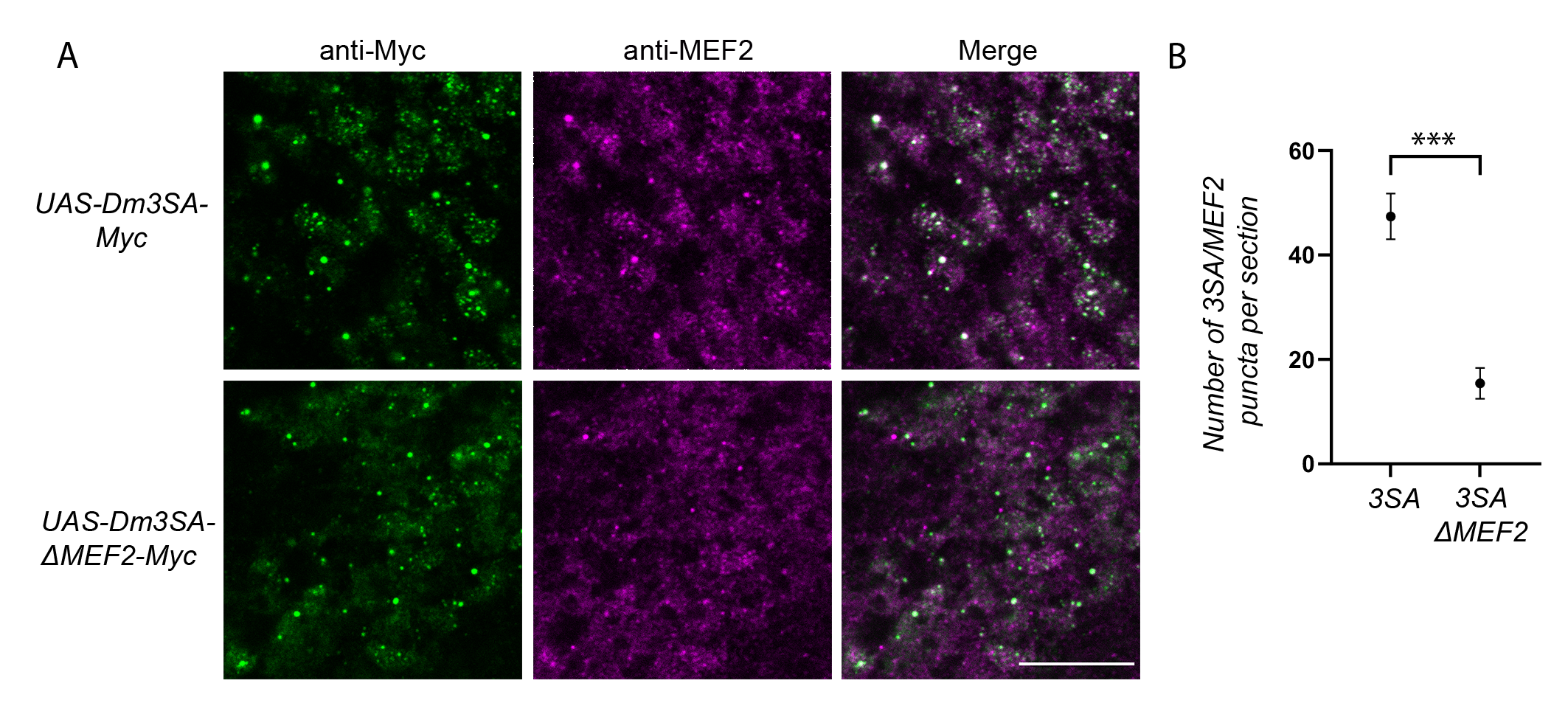

Supplement: Supplementary Figure 2 — Mutation of the MEF2 binding site reduces binding of 3SA to MEF2. OK107-GAL4; tub-GAL80ts females were crossed to UAS-Dm3SA and UAS-Dm3SA-ΔMEF2 males, and expression was induced in the brains of adult progeny by raising the flies at 18°C until after eclosion, then placing at 30°C for 72 h. A. Whole-mount brains were subjected to immunohistochemistry with anti-Myc (green) and anti-MEF2 (magenta). Z-stacks were captured of 0.25 μm slices, which were imaged at x100 magnification. Overlapping puncta appear white in the merged channel. B. The number of overlapping puncta in each section were counted using ImageJ in each of ∼40 sections through the Kenyon cell layer. Dm3SA-ΔMEF2 (n = 7 brains) displayed significantly reduced co-distribution with MEF2 in comparison to Dm3SA (n = 5 brains) [t-test t(10) = 6.318, p < 0.0001]. Scale bar = 10 μm. [file Image_2.TIF]
